# *Borrelia miyamotoi* Infection in Immunocompromised Man, California, USA, 2021

**DOI:** 10.3201/eid2905.221638

**Published:** 2023-05

**Authors:** Luis Alberto Rubio, Anne M. Kjemtrup, Grace E. Marx, Shanna Cronan, Christopher Kilonzo, Megan E.M. Saunders, Jamie L. Choat, Elizabeth A. Dietrich, Kelly A. Liebman, Sarah Y. Park

**Affiliations:** University of California, San Francisco, San Francisco, California, USA (L.A. Rubio);; California Department of Public Health, Sacramento, California, USA (A.M. Kjemtrup);; Centers for Disease Control and Prevention, Fort Collins, Colorado, USA (G.E. Marx, J.L. Choat, E.A. Dietrich);; County of Marin Department of Health and Human Services, San Rafael, California, USA (S. Cronan);; California Department of Public Health, Richmond, California, USA (C. Kilonzo, M.E.M. Saunders);; Marin-Sonoma Mosquito and Vector Control District, Cotati, California, USA (K.A. Liebman);; Karius, Incorporated, Redwood City, California, USA (S.Y. Park)

**Keywords:** Borrelia miyamotoi, tick-borne diseases, microbial cell-free DNA sequencing, immunocompromised host, relapsing tick-borne fever, Ixodes pacificus, bacteria, parasites, ticks, vector-borne infections, California, USA

## Abstract

Infection with *Borrelia miyamotoi* in California, USA, has been suggested by serologic studies. We diagnosed *B. miyamotoi* infection in an immunocompromised man in California. Diagnosis was aided by plasma microbial cell–free DNA sequencing. We conclude that the infection was acquired in California.

*Borrelia miyamotoi* is a relapsing fever spirochete; infection is recognized in Europe, Japan, and the northeastern United States as an emerging human infectious disease (*1*,*2*). First identified in Japan in 1995 in *Ixodes persulcatus* ticks, *B. miyamotoi* has since been detected in other species of *Ixodes* ticks, including *I. ricinus* in Europe, *I. scapularis* in eastern North America, and *I. pacificus* in western North America (*1*). In California, USA, *I*. *pacificus* ticks can harbor 2 spirochetes capable of causing human disease: *B. miyamotoi* and *Borrelia burgdorferi*, the agent that causes Lyme disease (*3*). Prevalence of *B. miyamotoi* in *I. pacificus* ticks in California is estimated to be 0.8% in adult ticks and 1.4% in nymphal ticks*,* similar to other parts of the world that have *Ixodes* spp. ticks and reported human cases of *B. miyamotoi* infection (*1*).

In California, tick-borne relapsing fever is usually ascribed to infection with *B. hermsii*, transmitted by soft ticks (*Ornithodoros hermsi*), found in high-elevation habitats (*4*). Although infection with *B. miyamotoi* in California has been suggested by serologic studies, clinical human cases of *B. miyamotoi* infection acquired in the western United States have not been reported in the literature (*1*,*3*,*4*). We describe *B. miyamotoi* infection, confirmed through plasma microbial cell–free DNA (mcfDNA) sequencing, in a California man with relapsing fevers. Our investigation was determined to be exempt from human subjects research by the Office of Human Research Protections of the California Health and Human Services Agency (Federalwide Assurance no. 00000681).

## The Case

In December 2021, an adult man receiving ocrelizumab (anti-B lymphocyte CD20 monoclonal antibody) for multiple sclerosis diagnosed in 2018 sought care at a neurology clinic in San Francisco, California, USA. The patient reported having experienced new fevers up to 38.7°C beginning in October 2021. The febrile episodes typically lasted 1 day, occurred every 10–14 days, and were associated with night sweats, mild vision changes, and nausea. Results of a physical examination were unremarkable, and the patient was sent home with a recommendation to return for evaluation if fever recurred.

Given continued intermittent fevers, the neurologist referred the patient to a local hospital in Greenbrae, California, USA, 3 days later for an expedited evaluation. Although the patient was again afebrile and results of physical examination were unremarkable, laboratory results were notable for thrombocytopenia (96,000 cell/mL [reference range 150,000–400,000 cells/mL]), elevated C-reactive protein level (47.2 mg/L [reference <5.0 mg/L]), and elevated procalcitonin level (1.89 ng/mL [reference <0.10 ng/mL]). No abnormalities were noted on chest radiographs or computed tomography scans of the abdomen and pelvis. Peripheral blood cultures were without growth, and the patient was discharged to home with a referral to the infectious disease clinic in San Francisco. At that clinic, serologic evaluation for certain bacteria, fungi, and viruses was notable only for positive Epstein-Barr viral capsid and nuclear antigen IgG. Serologic test results for *Borrelia burgdorferi*, brucellosis, and leptospirosis were negative, and results of a peripheral blood smear were unremarkable (Table 1).

**Table 1:** Laboratory test results for patient with *Borrelia miyamotoi* Infection, California, USA, 2021*

Blood test (reference values)	Dec 2021	May 2022
Leukocytes (4.0–11.0 k/uL)	5.1	4.8
Hemoglobin (13.5–18.0 g/dL)	13.3 (low)	15.2
Hematocrit (40.0%–52.0%)	38.5% (low)	43.4%
Thrombocytes (150–400 l/uL)	96,000 (low)	224,000
Sodium (136–145 mmol/L)	134 (low)	137
Creatinine (0.70–1.30 mg/dL)	0.82	0.98
Albumin (2.8–4.7 g/dL)	3.6	4.7
Aspartate transaminase (<40 U/L)	45 (high)	41 (high)
Alanine transaminase (<65 U/L)	46	46
Alkaline phosphatase (<38 U/L)	81	60
Total bilirubin (<1.0 mg/dL)	1.0	0.8
C-reactive protein (<5.0 mg/L)	47.2 (high)	0.2
Procalcitonin (<0.10 ng/mL)	1.89 (high)	<0.10
COVID-19/influenza/RSV PCR	Not detected	ND
Peripheral blood cultures (2 cultures in Dec 2022)	No growth at 5 days	ND
Hepatitis B surface Ab (>10 mIU/mL)	113	ND
Hepatitis B surface Ag	Nonreactive	ND
Hepatitis B core Ab	Nonreactive	ND
Hepatitis C Ab	Nonreactive	ND
EBV VCA IgM (<36 U/mL)	<36 U/mL	ND
EBV VCA IgG Ab (<18 U/mL)	248 (high)	ND
EBV EBNA IgG (<18 U/mL	255 (high)	ND
CMV IgM (<30 AU/mL)	<30	ND
CMV IgG (<0.6 U/mL)	<0.6	ND
*Cryptococcus* Ag	Not detected	ND
*Coccidioides* Ab, CF (<1:2)	<1:2	ND
*Coccidioides* Ab, ID	Negative	ND
*Histoplasma* Ab, CF (<1:8)	<1:8	ND
*Histoplasma*, ID	Negative	ND
*Histoplasma* Ag, urine (<0.2 ng/mL)	<0.2	ND
*Blastomyces* Ab, ID	Negative	ND
*Bartonella* Ab	Negative	ND
*Coxiella burnetti* Ab IgM and IgG	Negative	ND
*Rickettsia* Ab panel	Negative	ND
Syphilis (*Treponema*) screen by RPR (nonreactive)	Nonreactive	ND
Quantiferon-TB Gold Plus	Negative	ND

*Additional testing: February 2022, *Borrelia burgdorferi*, Brucella, and *Leptospira* Ab results all negative; March 2022, β-d-glucan (reference value <60 pg/mL), <31 pg/mL; galactomannan Ag (reference value <0.5), 0.018; *Coccidioides* Ag, serum, negative; sequencing of microbial cell–free DNA in plasma performed at Karius, Inc. (https://kariusdx.com) (reference value <10 DNA MPM), *Borrelia miyamotoi* 56 DNA MPM; relapsing fever PCR performed at CDC, *Borrelia* PCR positive group *B. miyamotoi* .Ab, antibody; Ag, antigen; CDC, Centers for Disease Control and Prevention; CF, complement fixation; CMV, cytomegalovirus; EBV, Epstein-Barr virus; EBNA, Epstein-Barr nuclear Ag; ID, immunodiffusion; MPM, molecules/mL; RPR, rapid plasma reagin; RSV, respiratory syncytial virus; VCA, viral capsid antigen.

Given the patient’s immunocompromised status and relapsing fever history, suspicion for infection remained high and plasma mcfDNA sequencing (Karius test, https://kariusdx.com) was ordered. Results were positive for *B. miyamotoi* (56 DNA molecules/μL [reference <10 molecules/μL]). The Centers for Disease Control and Prevention performed confirmatory *Borrelia* PCR testing, results of which were also positive for *B. miyamotoi* (*5*), and multilocus sequence typing (MLST), which indicated that the sequence was 100% identical in >6,040 nucleotides to a *B. miyamotoi* isolate from an *I. pacificus* tick collected in Marin County, California (Table 2) (*6*,*7*). The sequence was distinct from *B. miyamotoi* isolates from other geographic regions, displaying 99.3% identity (44 nt differences) to isolates from the eastern United States.

**Table 2:** Primers used to amplify genes for multilocus sequencing of *Borrelia miyamotoi* that infected immunocompromised man in California, 2021*

Gene	Forward primer, 3′ → 5′	Reverse primer, 5′ → 3′
*clpA*	TTGATCTCTTAGATGATCTTGG	CAAACATAAACCTTTTCAGCCTTTAATA
*clpX*	TTATCTGTTGCTGTTTATAATC	TTCAAACATAACATCTTTAAGTAATTCTTC
*nifS*	GAAAAAGTAAACTCCCTCAGAARGG	CAATGATGCCTGCAATATTTGGTG
*pepX*	AGAGACTTAAATTTAGCAGGAGTTG	TGCATTCCCCACATTGGAGTTC
*pyrG*	TTTAGTAATTGAGATTGGTGGTAC	TATTCCACAAACATTACGAGC
*recG*	TAGCATTCCTTTAGTTGAGGC	CTCAGCATGCTCAACTACC
*rplB*	AACTTATAGGCCAAAAACTTC	GATACAGGATGACGACCACC
*uvrA*	TTAAATTTTTAATTGATGTTGGACT	TCTGTAAAAAACCCAACATAAGTTGC

*Genes derived from (*6*), with modifications to the *nifS* and *rplB* forward primers to make them more specific for *B. miyamotoi*.

After *B. miyamotoi* infection was diagnosed, a 4-week course of doxycycline was prescribed. The patient reported having 1 additional febrile episode (38.9°C) after the first dose, but fevers subsequently resolved, and all other signs/symptoms improved. After consultation with the patient’s neurologist, cerebrospinal fluid testing was not performed, given resolution of visual symptoms and absence of other focal neurologic deficits. The patient returned to the clinic 1 month after having completed the course of doxycycline without any fever recurrence; laboratory testing showed resolution of thrombocytopenia and normalization of inflammatory markers. Ocrelizumab infusions were resumed after *B. miyamotoi* treatment, and symptom recurrence has not been reported.

The patient was a resident of Marin County, California, and reported having traveled 2 months before fever onset to Ohio and within California to Mendocino and Monterey Counties but reported no travel outside the United States for the previous 2 years. He reported hiking and swimming in freshwater lakes while traveling and near home but did not recall any insect or tick bites. He reported often spending time outdoors near home. He owned 2 domestic indoor cats not receiving regular tick prevention and reported no other animal exposures.

The California Department of Public Health Vector-Borne Disease Section collaborated with Marin-Sonoma Vector Control Agency to collect and test ticks from areas around the patient’s residence. The habitat consisted of coastal redwood grove and understory grass and shrub vegetation. Questing ticks were collected by dragging a 1-m^2^ white flannel cloth along the ground cover. A total of 19 *I. pacificus* ticks (12 adults, 7 nymphs) from the patient’s yard and nearby trails were collected and stored in 70% ethanol until DNA extraction. PCR testing did not identify any ticks positive for *B. miyamotoi* infection but identified 1 adult tick infected with *B. burgdorferi* sensu lato (*8*).

## Conclusions

Although *B. miyamotoi* has been identified in ticks in California for >20 years, locally acquired human cases within the western United States have not been described (*1*). Our environmental investigation identified multiple *I. pacificus* ticks near this patient’s residence and recreation areas in California, all in locations where *B. miyamotoi* has been documented in *I. pacificus* ticks (*1*). The *B. miyamotoi* sequence recovered from the patient was most closely related to an isolate recovered from an *I. pacificus* tick in California (*7*). Of note, the patient’s travel to Ohio was during August, when seasonal activity of *Ixodes* spp. ticks in the region is low, and was to a location where *B. miyamotoi* has not been identified (*9*).

For patients with high or relapsing fever during seasons of *Ixodes* tick activity, particularly in areas where *B*. *miyamotoi* has been reported in local tick populations, clinicians should consider the possibility of *B. miyamotoi* infection along with other *Borrelia* spp. Laboratory confirmation of *B. miyamotoi* infection can be challenging because the spirochetes share many proteins with *B. burgdorferi* and *B. hermsii*, resulting in cross-reacting antibodies, and because few laboratories offer specific molecular diagnostic testing for *B. miyamotoi* (*10*). For this case, *B. miyamotoi* infection was diagnosed through molecular testing with unbiased plasma mcfDNA sequencing, an increasingly used tool for evaluating patients with fever of unknown etiology (*11*,*12*). The patient’s immunocompromised status may have contributed to the infection chronicity, increasing our ability to detect the organism (*11*,*13*). PCR and sequencing confirmed the diagnosis.

Our study suggests that *B. miyamotoi* is an emerging human pathogen in California. Human infection is probably rare, given low seroprevalence in blood donors, even in counties to which *I. pacificus* ticks are endemic, and low prevalence of *B. miyamotoi* in ticks that is rarely >2% (*1*,*3*). Given limitations of serologic testing, clinicians should maintain an index of suspicion for *B. miyamotoi* in patients with relapsing fever without a clear etiology, should ask about potential tick exposure, and should consider molecular diagnostic testing.

## References

[1] Padgett K, Bonilla D, Kjemtrup A, Vilcins IM, Yoshimizu MH, Hui L, et al. Large scale spatial risk and comparative prevalence of *Borrelia miyamotoi* and *Borrelia burgdorferi* sensu lato in *Ixodes pacificus.* PLoS One. 2014;9:e110853. 10.1371/journal.pone.0110853 25333277 PMC4205013

[2] Sato K, Takano A, Konnai S, Nakao M, Ito T, Koyama K, et al. Human infections with *Borrelia miyamotoi*, Japan. Emerg Infect Dis. 2014;20:1391–3. 10.3201/eid2008.131761 25061761 PMC4111186

[3] Brummitt SI, Kjemtrup AM, Harvey DJ, Petersen JM, Sexton C, Replogle A, et al. *Borrelia burgdorferi* and *Borrelia miyamotoi* seroprevalence in California blood donors. PLoS One. 2020;15:e0243950. 10.1371/journal.pone.0243950 33370341 PMC7769429

[4] Krause PJ, Carroll M, Fedorova N, Brancato J, Dumouchel C, Akosa F, et al. Human *Borrelia miyamotoi* infection in California: Serodiagnosis is complicated by multiple endemic *Borrelia* species. PLoS One. 2018;13:e0191725. 10.1371/journal.pone.0191725 29420552 PMC5805228

[5] Dietrich EA, Replogle AJ, Sheldon SW, Petersen JM. Simultaneous detection and differentiation of clinically relevant relapsing fever *Borrelia* with semimultiplex real-time PCR. J Clin Microbiol. 2021;59:e0298120. 10.1128/JCM.02981-20 33910966 PMC8218771

[6] Margos G, Gatewood AG, Aanensen DM, Hanincová K, Terekhova D, Vollmer SA, et al. MLST of housekeeping genes captures geographic population structure and suggests a European origin of *Borrelia burgdorferi.* Proc Natl Acad Sci U S A. 2008;105:8730–5. 10.1073/pnas.0800323105 18574151 PMC2435589

[7] Kingry LC, Replogle A, Dolan M, Sexton C, Padgett KA, Schriefer ME. Chromosome and large linear plasmid sequences of a *Borrelia miyamotoi* strain isolated from *Ixodes pacificus* ticks from California. Genome Announc. 2017;5:e00960–17. 10.1128/genomeA.00960-17 28912318 PMC5597759

[8] Barbour AG, Bunikis J, Travinsky B, Hoen AG, Diuk-Wasser MA, Fish D, et al. Niche partitioning of *Borrelia burgdorferi* and *Borrelia miyamotoi* in the same tick vector and mammalian reservoir species. Am J Trop Med Hyg. 2009;81:1120–31. 10.4269/ajtmh.2009.09-0208 19996447 PMC2841027

[9] Krause PJ, Fish D, Narasimhan S, Barbour AG. *Borrelia miyamotoi* infection in nature and in humans. Clin Microbiol Infect. 2015;21:631–9. 10.1016/j.cmi.2015.02.006 25700888 PMC4470780

[10] Fleshman AC, Foster E, Maes SE, Eisen RJ. Reported county-level distribution of seven human pathogens detected in host-seeking *Ixodes scapularis* and *Ixodes pacificus* (Acari: Ixodidae) in the contiguous United States. J Med Entomol. 2022;59:1328–35. 10.1093/jme/tjac049 35583265

[11] Wright WF, Simner PJ, Carroll KC, Auwaerter PG. Progress report: next-generation sequencing, multiplex polymerase chain reaction, and broad-range molecular assays as diagnostic tools for fever of unknown origin investigations in adults. Clin Infect Dis. 2022;74:924–32. 10.1093/cid/ciab155 33606012

[12] Haidar G, Singh N. Fever of unknown origin. N Engl J Med. 2022;386:463–77. 10.1056/NEJMra2111003 35108471

[13] Boden K, Lobenstein S, Hermann B, Margos G, Fingerle V. *Borrelia miyamotoi*–associated neuroborreliosis in immunocompromised person. Emerg Infect Dis. 2016;22:1617–20. 10.3201/eid2209.152034 27533748 PMC4994329

